# *Vitis vinifera* L. Fruit Diversity to Breed Varieties Anticipating Climate Changes

**DOI:** 10.3389/fpls.2018.00455

**Published:** 2018-05-01

**Authors:** Antoine Bigard, Dargie T. Berhe, Eleonora Maoddi, Yannick Sire, Jean-Michel Boursiquot, Hernan Ojeda, Jean-Pierre Péros, Agnès Doligez, Charles Romieu, Laurent Torregrosa

**Affiliations:** ^1^AGAP, University of Montpellier, CIRAD, INRA, Montpellier SupAgro, Montpellier, France; ^2^UE INRA de Pech-Rouge, University of Montpellier, INRA, Montpellier, France; ^3^SNNPRS, Dilla University, Dilla, Ethiopia; ^4^UE INRA de Vassal, Grapevine Biological Resource Centre, University of Montpellier, INRA, Montpellier, France

**Keywords:** climate warming, fruit growth, sugars, organic acids, genetics, grapevine, *Vitis vinifera*

## Abstract

The wine industry is facing critical issues due to climate changes since production is established on very tight Genotype × Environment interaction bases. While, some cultivation practices may reduce adverse effects of abiotic stresses on the vines, e.g., the use of irrigation to mitigate drought, the deleterious impacts of warming on fruit development are difficult to manage. Elevated temperature alters grapevine fruit growth and composition, with a critical increase of the sugars/organic acids ratio. Select grapes with improved metabolite balances to offset high temperature effects is a valuable option to sustain viticulture. Unfortunately, the lack of knowledge about the genetic diversity for fruit traits impacted by temperature impairs the design of breeding programs. This study aimed to assess the variation in berry volume, main sugars and organic acids amounts in genetic resources. Fruit phenotyping focused on two critical stages of development: the end of green lag phase when organic acidity reaches its maximum, and the ripe stage when sugar unloading and water uptake stop. For that purpose, we studied a panel of 33 genotypes, including 12 grapevine varieties and 21 microvine offspring. To determine the date of sampling for each critical stage, fruit texture and growth were carefully monitored. Analyses at both stages revealed large phenotypic variation for malic and tartaric acids, as well as for sugars and berry size. At ripe stage, fruit fresh weight ranged from 1.04 to 5.25 g and sugar concentration from 751 to 1353 mmol.L^-1^. The content in organic acids varied both in quantity (from 80 to 361 meq.L^-1^) and in composition, with malic to tartaric acid ratio ranging from 0.13 to 3.62. At the inter-genotypic level, data showed no link between berry growth and osmoticum accumulation per fruit unit, suggesting that berry water uptake is not dependent only on fruit osmotic potential. Diversity among varieties for berry size, sugar accumulation and malic to tartaric acid ratio could be exploited through cross-breeding. This provides interesting prospects for improving grapevine to mitigate some adverse effects of climate warming on grapevine fruit volume and quality.

## Introduction

With 75–85 million tons of grapes produced yearly in the world, the grapevine is the main fruit crop^1^^,^^2^. Grapevine fleshy berry, classified as a non-climacteric fruit (Coombe, 1976), undergoes a complex development process including two growth phases (Mullins et al., 1992). The first growth phase results from cell division and expansion coupled with the accumulation of organic acids, mainly tartrate and malate (Kliewer, 1965). After a lag phase called green plateau, fruit softens and massive uptake of sugars triggers a second phase of flesh cell enlargement (Matthews et al., 1987). Considering their sequential accumulation, organic acids (up to 250 mmol.L^-1^) and sugars (up to 1 M) appear as the main drivers of berry osmotic potential during green and ripening growth phases, respectively. Other solutes, such as potassium, which only peaks at 30 mmol.L^-1^ at ripe stage, would be minor players in fruit osmotic potential (Rogiers et al., 2017). The final concentrations of sugars and organic acids at ripe stage determine the ethanol to acidity ratio after yeast fermentation, which is a primary factor of wine quality (Champagnol, 1984; Ribéreau-Gayon et al., 2006).

Domesticated *Vitis vinifera* L., the major grapevine species cultivated for wine production, is supposed to have been diffused from the South Caucasus toward Mediterranean regions (This et al., 2006; Bacillieri et al., 2013), using a little fraction of the genetic diversity present in this species (Myles et al., 2011; Zhou et al., 2017). Modern wine, juice and table grape industries only use a limited number of *V. vinifera* cultivars (Wolkovich et al., 2018) which are established in very tight interactions with climatic conditions and cultivation practices (Carbonneau et al., 2015). In 2016, the first 30 *V. vinifera* cultivars represented 85% of the plant material released by French nurseries, with the top 10 genotypes accounting for more than 65% of the production^3^. In traditional European vine growing regions, as well as in more recently developed areas (United States, Australia, China), only a few elite cultivars are planted that represents a small fraction of the grapevine germplasm (Galet, 2000; Goldammer, 2015; Wolkovich et al., 2018).

Climate change has already induced noticeable changes in the grapevine development cycle and wine composition (Ganichet, 2002; Seguin et al., 2004; Duchêne and Schneider, 2005; Drappier et al., 2017; Ojeda et al., 2017a). Current models anticipate a further increase from +2°C to +5°C within a few decades (Bock et al., 2013; Fraga et al., 2013; Hannah et al., 2013), which represents a serious threat for wine production in several regions. The impact of environmental factors has been studied on grapevine vegetative or reproductive organs (Butrose, 1969a,b; Webb et al., 2007; Greer, 2012; Coupel-Ledru et al., 2014; Xu et al., 2014; Luchaire et al., 2017) and fruit composition (for a review, see Dai et al., 2011). Butrose et al. (1971) reported that the increase in temperature decreased berry size while increasing sugar concentration. Elevated temperature has been shown to reduce malic acid (Butrose et al., 1971; Lakso and Kliewer, 1978; Sweetman et al., 2014) and anthocyanidin contents in berries (Kliewer and Torres, 1972; Mori et al., 2007). In the last 15 years, the molecular regulation of the synthesis and transport of main primary and secondary metabolites in the grapevine has received considerable attention (Terrier et al., 2001; DeBolt et al., 2006; Hichri et al., 2011; Rienth et al., 2016b). The first process-based models of metabolite accumulation in grapevine fruit have only recently been established (Dai et al., 2013; Vivin et al., 2017).

Changing cultural practices is the first option to reduce adverse climatic effects (Van Leeuwen et al., 2013). For instance, watering is a very efficient measure to mitigate drought (Ojeda et al., 2002). However, the effects of heat stress on berry development and composition are more difficult to control. Several attempts were made to decrease the rate of sugar accumulation into the berry, e.g., using anti-transpirant sprays or leaf removal to reduce carbon assimilation (Gatti et al., 2016a), shading nets to decrease photosynthetic capacity (Greer et al., 2011), minimal pruning to change vine canopy structure (Martínez De Toda et al., 2015). Some of these practices were found effective to reduce sugar accumulation, but with deleterious effects on vegetative growth and secondary metabolite accumulation into fruits (Greer et al., 2011; Bobeica et al., 2015). Delaying winter pruning to shift berry development toward cooler periods in the autumn (Ravaz, 1912; Gatti et al., 2016b) was found irrelevant. Since none of these adaptations proved efficient enough to offset the expected changes in temperature, a promising alternative could be to take advantage of the grapevine genetic diversity to select grapes with improved developmental and metabolic properties (Ollat et al., 2014; Torregrosa et al., 2017a).

Phenotypic variability, which is an intrinsic property of all species, results from genetic (G), environment (E) or GxE interactions (Conde et al., 2007). Wolkovich et al. (2018) recently claimed that enough genetic diversity exists in *V. vinifera* phenology to mitigate the adverse effects of climate warming on grapes quality. However, Ollat et al. (2015) showed that late ripening cultivars from southern European regions are inefficient to compensate the ripening time shifts that are expected in Bordeaux region. Indeed, Xinomavro from Greece, or Carignan from Spain would even ripe earlier than Petit Verdot, which is already used in Bordeaux wines. While climate models anticipate an phenology advance of several weeks, the latest varieties experimented by Ollat et al. (2015) only ripen a few days later than Cabernet-Sauvignon, the emblematic variety of Bordeaux. Moreover, the effects of global warming on the composition of the grape at harvest can not only be analyzed on the acceleration of reproductive development since water, metabolites and inorganic compounds into the fruit are differentially impacted by temperature (Kliewer and Lider, 1970; Kliewer and Torres, 1972; Barnuud et al., 2014).

Therefore, there is an urgent need to evaluate the grapevine diversity for berry development and composition (Gascuel et al., 2017), focusing on attributes that are impacted by temperature, i.e., the berry volume and the accumulation of sugars, organic acids and secondary metabolites. Few studies exist on the diversity of grape composition in *V. vinifera* germplasm (Shiraishi et al., 2010; Houel et al., 2013; Preiner et al., 2013; Teixeira et al., 2013; Yinshan et al., 2017) or in breeding populations (Doligez et al., 2006, 2013; Liu et al., 2006, 2007; Mejia et al., 2007; Duchêne et al., 2012, 2013; Chen et al., 2015; Costantini et al., 2015; Houel et al., 2015). Unfortunately, in most of these studies, fruit developmental stages were ambiguously defined and berry parameters were characterized independently one from each other, resulting in some confusion between water and metabolites accumulation or concentration. In this study, we have measured at the same time the main berry traits that could vary with temperature increase in 33 *V. vinifera* genotypes. The whole genotype set consisted in a first subset of wine grape cultivars and a second subset of microvine offspring, this latter model being very promising for both physiological and genetic studies (Chaib et al., 2010; Rienth et al., 2016b; Luchaire et al., 2017; Sanchez-Gomez et al., 2017). The phenotypic diversity for growth and solutes accumulation was characterized at two critical stages of grapevine fruit development: (i) the end of green growth phase, when the berry stops loading organic acids and (ii) the end of ripening, when the contents of water and sugars reach their maximum.

## Materials and Methods

### Plant Material and Growing Conditions

**FIGURE 1:**
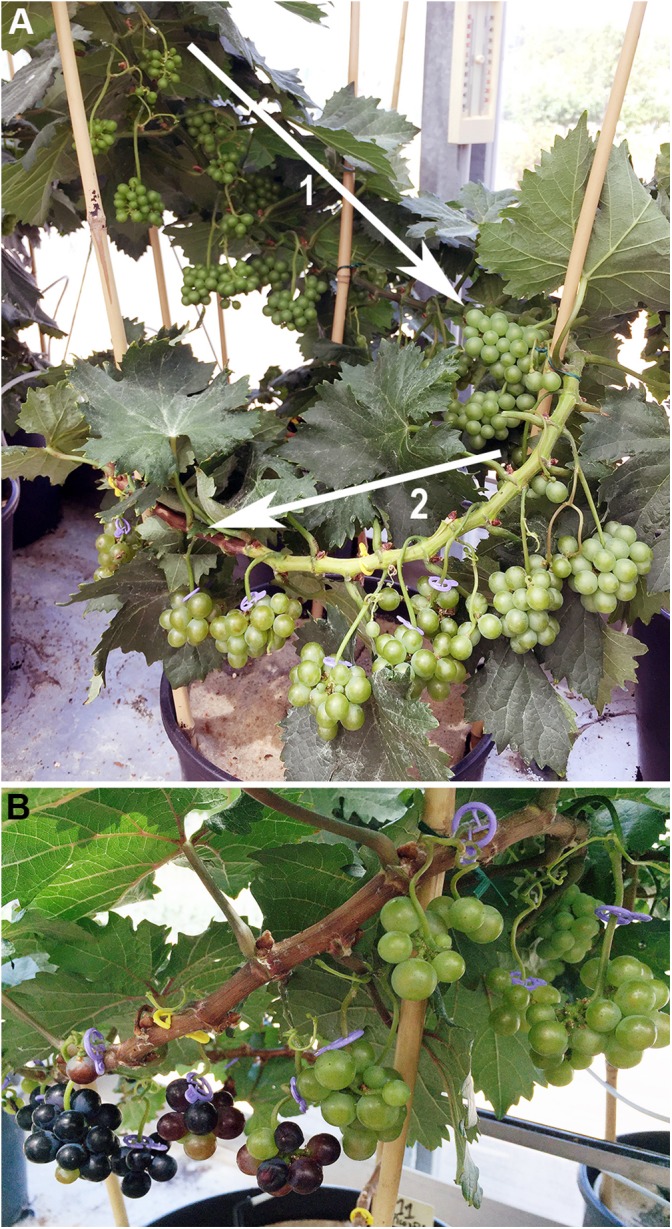
The typical continuous fruit development along a microvine proleptic shoot. **(A)** The offspring n°98 displaying non-pigmented fruits during green (arrow 1) and ripening (arrow 2) growth stages. **(B)** The onset of ripening (*véraison*) as it is observable on the offspring n°11 which develops anthocyanidin-pigmented berries.

Based on expert’s advice and preliminary experiments, all genotypes included in this study displayed contrasted features for berry size and soluble solid contents at ripening. The first subset of genotypes consisted in 12 *V. vinifera* varieties (Supplementary Table S1). In 2016, the 12 *V. vinifera* varieties were phenotyped at the Grapevine Biological Resources Centre (GBRC) of Vassal (Marseillan, France), where the vines were grown in sandy soils as ungrafted and non-irrigated plants (Experiment 1). In 2017, the phenotyping was repeated for six of the varieties that were present on the grapevine collection of Montpellier SupAgro Campus (Montpellier, France). In this collection, which was established from the GBRC 15 years ago, the vines were grown in gravelly soils as grafted and fertirrigated plants (Experiment 2). In both experiments, each variety was established as 5–20 replicated plants managed by spur pruning with vertical shoot positioning (VSP). To avoid the effects of source/sink unbalance, the number of clusters was reduced to 4–8 per vine after berry set. The second subset included 21 offspring of microvines from a cross between the Picovine00C001V0008 (*Vvgai1*/*Vvgai1*), which confers to the progeny Dwarf and Rapid Cycling and Flowering (DRCF) traits (Chaib et al., 2010), and the Ugni Blanc fleshless berry mutant (*flb*; Fernandez et al., 2006b). Microvine phenotypes were recorded in two experiments performed in two different greenhouses. In 2016 (Experiment 3), two replicates of 4-years-old own-rooted potted plants for each of the 21 microvine offspring (Supplementary Table S1) were established at the INRA experimental unit of Pech-Rouge (Gruissan, France). In 2017 (Experiment 4), 2–4 replicates of 3–5 years-old own-rooted potted plants for six microvines offspring were established at the Montpellier SupAgro Campus (Montpellier, France). In both experiments, night/day temperatures were maintained at 15/25 ± 5°C and the microvines were watered at full PET (potential evapotranspiration). To standardize vegetative and reproductive development of the microvines, lateral branches were systematically removed as described by Luchaire et al. (2017), to keep a single proleptic shoot per plant (**Figure 1**). The experiments for varieties and microvines were performed in different environmental contexts to appreciate the stability of the phenotypes. For varieties, main changes between Experiments 1 and 2, corresponded to grafting, watering, soil type, exposition and temperatures (Supplementary Table S2). For microvines, main changes between Experiments 3 and 4, corresponded to the plant age and air temperature (Supplementary Table S2). Thus, in the rest of the manuscript, the terms experiments, environment or year are indifferently used.

### Fruit Sampling Methods

In Experiment 1, starting before fruit softening, nine clusters per varieties were monitored weekly for single berry firmness. When the first soft berries was detected, 4–30 hard green berries were sampled to represent the stage with the highest contents in organic acids. For each of the nine clusters, 2–54 berries were sampled 3, 4, and 5 weeks later. In Experiment 2, first sampling date was determined as 2016 with a higher precision and only clusters presenting both hard and soft berries were maintained in plants to address synchronized bunches. To gain in the accuracy of the determination of ripe stage, two clusters per variety were immersed 3 times a week to non-destructively monitor the evolution of berry volume as described in Torregrosa et al. (2008). Several samples were collected at 3-day intervals when berry growth started to slow down. All samplings were performed in triplicate (3 × 30 berries). In Experiment 3, 2–11 microvine hard berries were sampled from individual clusters with the same procedure as described above. Berry firmness was manually assessed twice a week to identify which cluster displayed the first signs of berry softening, and 2–13 berries were then sampled on each of the two clusters above. Thanks to the continuous production of clusters in microvines, at least three replicates were collected at 1- to 2-weeks intervals from each plant for each developmental stage. In Experiment 4, microvine plants were grown up to simultaneously display all reproductive stages from flowering to berry shriveling. For each plant replicate, 5–8 berries were systematically sampled on clusters present between 3 and 5 levels above the first bunch showing berry softening and 3-5 levels below the onset of berry shriveling. Berries of the same clusters were pooled for biochemical analyses, except for clusters at the onset of ripening (i.e., presenting both hard and soft berries) for which 5–8 single berries were separately analyzed. This allowed a precise selection of samples corresponding to the last stages of green berry development and maximum berry volume. For all genotypes, when the berry volume from successive clusters was very close or irregular, we selected the cluster displaying the maximum of sugar contents per berry and the lowest concentration in tartaric acid, assuming that it corresponded to the arrest of sugar unloading and water uptake.

### Berry Growth and Composition Determination

For Experiments 1 and 3, fresh berries were ground with a mortar and pestle at room temperature and stored at -30°C. To complete extraction and dissolve organic salts, samples were heated at 60°C for 30 min, vortexed during 30 s and then centrifuged at 18,500 *g* during 5 min at 20°C. Clear juice was diluted 10 times in 0.2 N HCl, and then filtered with sterile, non-pyrogenic, hydrophilic cellulose acetate 0.2 μm membranes before HPLC injection. In Experiments 2 and 4, we performed a new protocol that was validated in preliminary experiments to simplify primary metabolite extraction (data not shown). Single or pooled berries were added with 5X fresh weight of 0.25 N HCl. After 48 h incubation at room temperature, samples were diluted 10 times with 8.3 10^-3^ N acetic acid (internal control) + 16.4 10^-3^ N sulphuric acid. After centrifuging as above, supernatants were directly injected for HPLC to separate glucose, fructose, malic and tartaric acids through a Biorad aminex-HPX87H column according to Bories et al. (2011) with slight modifications (60°C and 0.6 ml.min^-1^ rate flow).

### Data Presentation and Statistical Analyses

**FIGURE 2:**
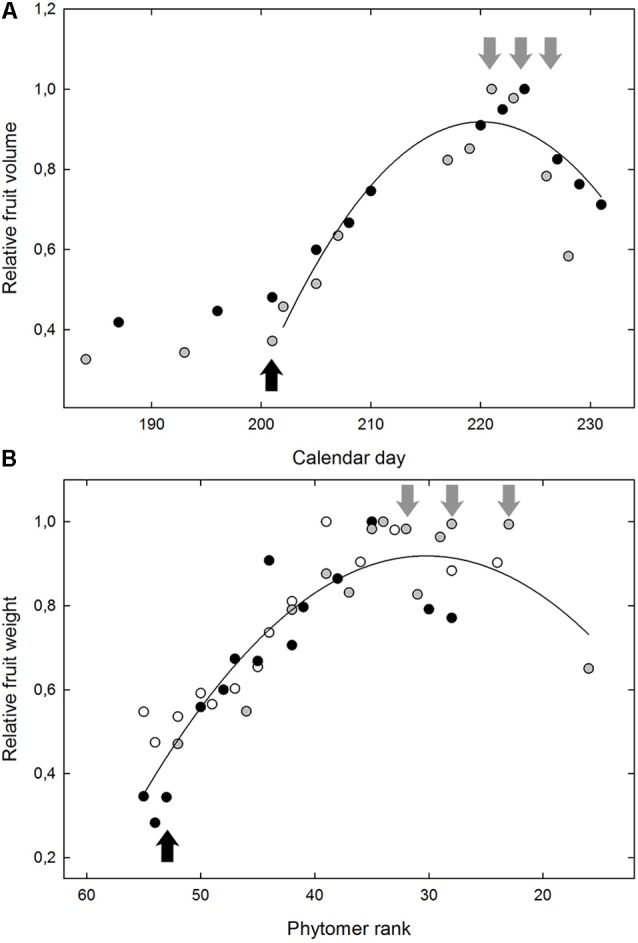
The typical fruit growth from onset of ripening for the Grenache variety **(A)** in Experiment 2 and for the microvine offspring n°114 **(B)** in Experiment 4. In **(A)**, the data correspond to the evolution of the relative fruit volume, as a function of the calendar day, with the maximum berry volume as 1. The average berry volume was non-destructively monitored by the immersion of 2 reference clusters (gray and black dots). In **(B)**, the relative berry weight is represented for 3 replicate plants of the microvine n°114 (gray, black, and white dots) as a function of the phytomer position from the base of the main shoot, with the maximum average berry weight as 1. Black arrows indicate the date/position of the samples for green berry. Gray arrows indicate the 3 dates/positions of the samples analyzed for ripe berries.

Except for **Figure 2**, presented data corresponded to targeted fruit developmental stages: the last stages of green berry development and the maximum volume of the berries. Statistical analyses for G, E and GxE interactions, were performed with R-software version 3.4.3 (R Core Team, 2017) on the six varieties and six microvine genotypes experimented in two environments. Pearson correlations were calculated between variables with interception to 0 (type of regression expected). The slope of the regressions was used to compare environmental effects. For mean comparisons, several tests were used depending on homoscedasticity pre-tests. Parametric Student’s *t*-test (one parameter) or ANOVA I and II (G, E, GxE interaction) were performed to data displaying a normal distribution and equal variance between treatments. Otherwise, non-parametric Wilcoxon (one parameter) and two-way ordinal regression (G, E, GxE) were performed. For classification tests, a comparison of least-square means at a 0.05 significance level and a Tukey adjustment was performed (Supplementary Tables S3, S4). Raw data and R codes will be provided upon request.

## Results

### Berry Growth During Ripening

**FIGURE 3:**
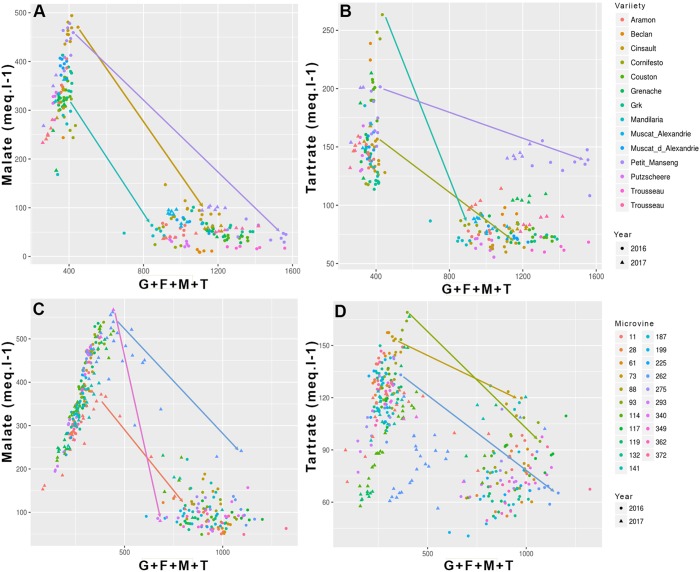
Evolution of the main acids (malic and tartaric) concentration as a function of sum of major osmoticum (glucose + fructose + malate + tartrate) concentration during ripening for variety **(A,B)** and microvine **(C,D)** subsets. Arrows show several contrasted trends for the evolution of malate and tartrate concentrations from the onset of ripening to ripe stage.

All varieties displayed similar kinetics of fruit growth, regardless of the large variation observed for berry volume at both green and ripe stages. Likewise, microvine fruits followed the same developmental trends as a function of the position along the primary shoot (**Figure 2**). The quantity of sugar accumulated per berry did not increase any longer in the two samples following maximum fruit volume (data not shown). Following maximum fruit volume, sugar concentration (or °Brix) increased through water loss, i.e., decrease in fruit volume, which may be marked for some genotypes. The contents in main metabolites considerably varied within genotypic subsets and samples, with a clear distinction between the two targeted stages of fruit development (**Figure 3**).

#### Berry Size

**FIGURE 4:**
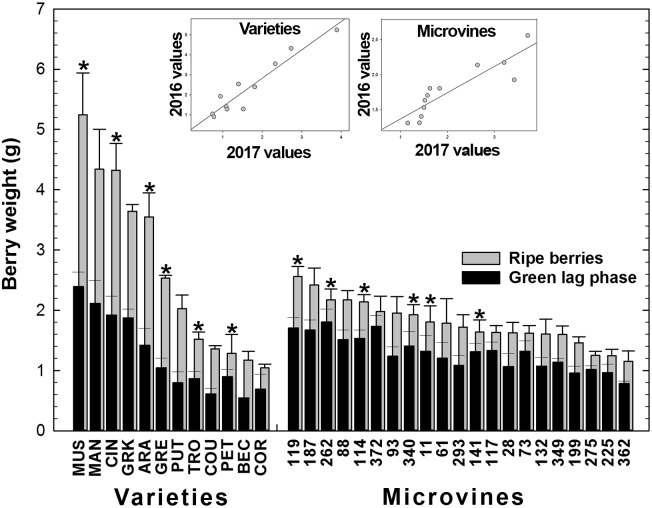
Diversity for the berry weight at the end of green growth and at ripe stage in varieties and microvine subsets. Bar chart represent 2016 mean values with the corresponding SE. Contrasted genotypes experimented in 2016 and 2017 are indicated by an asterisk. Inserted plots show the relationships between the mean values of both years (Supplementary Tables S3, S4 for detailed numeric values and statistics).

For varieties, in Experiment 1 (2016), berry weight ranged 1.04–5.25 g/berry at maximum berry volume (**Figure 4** and Supplementary Tables S3, S4), increasing on average by 2.10 ± 0.36 between green lag phase and ripe stage, with a coefficient of correlation of 0.97 (*p*-value = 6.53 10^-7^). In 2017 (Experiment 2), the increment in weight between green and ripe stage was similar (2.10 ± 0.53), with a coefficient of correlation of 0.92 between stages (*p*-value = 9.81 10^-3^). The increase of berry weight during ripening ranged from 1.4 for Petit Manseng to 2.9 for Cinsaut. In the microvine progeny, 2016 berry weight (Experiment 3) ranged from 1.15 to 2.56 g/berry at maximum berry volume, increasing by 1.39 ± 0.13 between the two stages, with a coefficient of correlation of 0.89 (*p*-value = 8.37 10^-8^). In 2017 (Experiment 4), the increase of berry weight during ripening was 1.84 ± 0.47 with a coefficient of correlation of 0.81 (*p*-value = 5.25 10^-2^). This increment ranged from 1.15 to 2.4, and was not correlated to maximum berry volume. The plots inserted in **Figure 4** show the year-to-year relationships for the six varieties and six microvines reproduced in 2017 (Supplementary Tables S3, S4). Statistical analyses showed a significant effect of genotype, environment and GxE interaction on both green and ripe berry weights for varieties and only on ripe berry weight for microvines. For microvine green fruit weight, only the effect of genotype was found statistically significant.

### Organic Acids

**FIGURE 5:**
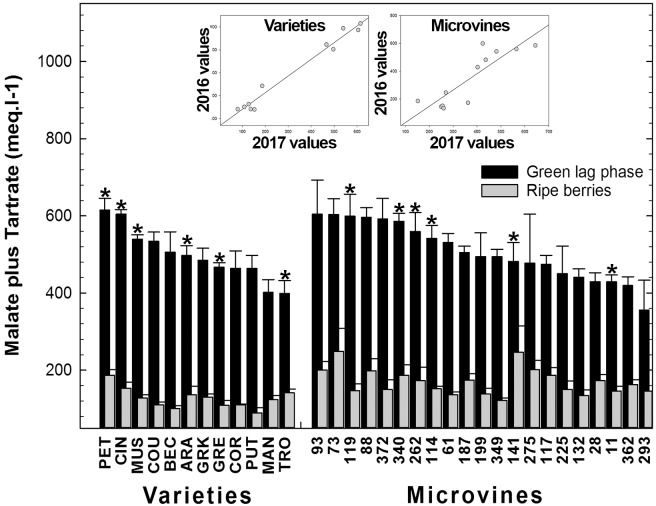
Diversity for the sum of malic and tartaric acid fruit concentrations at the end of green growth and at ripe stage in varieties and microvine subsets. Bar chart represent 2016 mean values with the corresponding SE. Contrasted genotypes experimented in 2016 and 2017 are indicated by an asterisk. Inserted plots show the relationships between the mean values of both years (Supplementary Tables S3, S4 for detailed numeric values and statistics).

**FIGURE 6:**
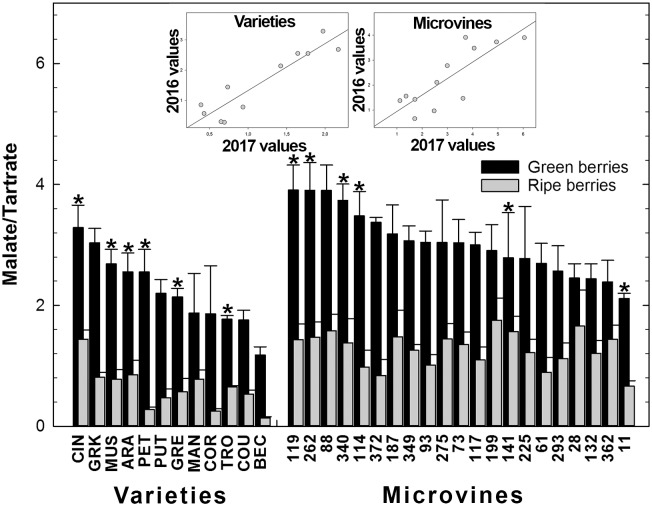
Diversity in the ratio malate/tartrate of the fruit at the end of green growth and at ripe stage in varieties and microvine subsets. Bar chart represent 2016 mean values with the corresponding SE. Contrasted genotypes experimented in 2016 and 2017 are indicated by an asterisk. Inserted plots show the relationships between the mean values of both years (Supplementary Tables S3, S4 for detailed numeric values and statistics).

Among all genotypes, in both years, the total concentration of malic and tartaric acids ranged from 401 to 644 meq.L^-1^ at the end of green growth phase and from 75 to 362 meq.L^-1^ at maximum berry volume (**Figure 5** and Supplementary Tables S3, S4). At ripe stage, the malate concentrations varied from 12 to 99 meq.L^-1^ among varieties and from 57 to 276 meq.L^-1^ among microvines (**Figures 3A,C**). The tartrate concentration varied from 60 to 146 meq.L^-1^ among varieties and from 51 to 114 meq.L^-1^ among microvines (**Figures 3B,D**) and such concentrations at ripe stage were higher in 2017 (Supplementary Table S4). For varieties, tartaric acid concentration between green lag phase and maximum berry volume decreased by 2.09 ± 0.43 in Experiment 1 and 1.67 ± 0.26 in Experiment 2. A significant correlation between this decrease and berry growth was observed in Experiment 2 (0.83, *p*-value 3.70 10^-2^). The malate/tartrate ratio ranged from 1.42 to 6.05 at the end of green growth stage and 0.14 to 3.62 at ripe stage (**Figure 6** and Supplementary Tables S3, S4). For varieties, this ratio was correlated with berry size at green stage with a correlation coefficient of 0.75 (*p*-value = 3.49 10^-4^) in 2016 (Experiment 1) and 0.68 (*p*-value = 4.68 10^-7^) in 2017 (Experiment 2).

The plots inserted in **Figures 5**, **6** show the year-to-year relationships for the six varieties and six microvines reproduced in 2017 (Supplementary Tables S3, S4). Statistical analyses showed a significant effect of genotype, environment and GxE interaction in both genotype subsets for the total acids content at ripe stage, but no environmental effect for microvine green fruits. For the malate/tartrate ratio at ripe stage, we found a significant effect of genotype, environment and GxE interaction in both genotype subsets for green fruits but no environmental effect in ripe fruits for varieties.

### Sugars

**FIGURE 7:**
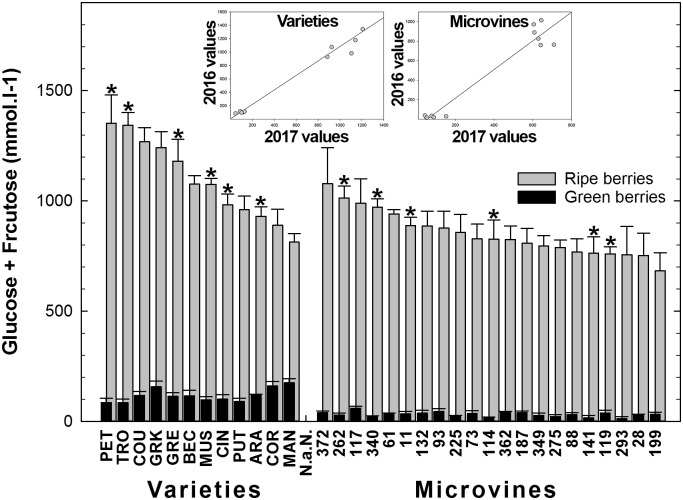
Diversity in the sum of glucose and fructose concentrations in fruit at the end of green growth and at ripe stage in varieties and microvine subsets. Bar chart represent 2016 mean values with the corresponding SE. Contrasted genotypes experimented in 2016 and 2017 are indicated by an asterisk. Inserted plots show the relationships between the mean values of both years (Supplementary Tables S3, S4 for detailed numeric values and statistics).

Among all genotypes, in both years, the Glucose + Fructose concentration varied from 12 to 153 mmol.L^-1^ at green lag phase, to 752–1353 mmol.L^-1^ at ripe stage (**Figure 7** and Supplementary Tables S3, S4) with higher average concentrations in varieties but no correlation was found between developmental stages. Correlations between sugar concentration at ripe stage and maximum berry volume was observed for varieties (-0.75 in 2016 and -0.54 in 2017). The rate of sugar accumulation during ripening ranged from 25 to 52 mmol.L^-1^.day^-1^. The plots inserted in **Figure 7** show the year-to-year relationships for the six varieties and six microvines reproduced in 2017 (Supplementary Tables S3, S4). Statistical analyses showed a significant effect of genotype, environment and GxE interaction in both genotype subsets for sugars contents at ripe stage, but no environmental effect in green fruits for varieties.

#### Osmoticum Accumulation

**FIGURE 8:**
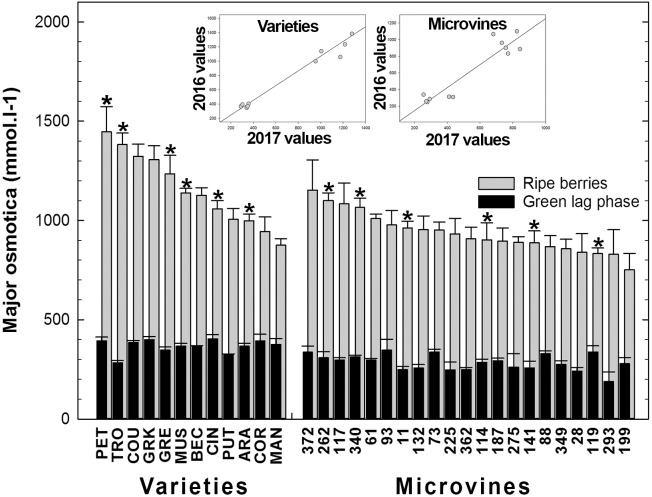
Diversity in the sum of major fruit osmotica (glucose, fructose, malic and tartaric acids) concentrations at the end of green growth and at ripe stage in varieties and microvine subsets. Bar chart represent 2016 mean values with the corresponding SE. Contrasted genotypes experimented in 2016 and 2017 are indicated by an asterisk. Inserted plots show the relationships between the mean values of both years (Supplementary Tables S3, S4 for detailed numeric values and statistics).

Among all genotypes, in both years, the total of main osmotica (Glucose + Fructose + Malate + Tartrate) varied from 190 to 436 mmol.L^-1^ at green lag phase to 605–1446 mmol.L^-1^ at maximum berry volume (**Figure 8** and Supplementary Tables S3, S4). Maxima for malic and tartaric acid concentrations were observed in green berries (**Figure 3**). At this stage, organic acids accounted for the main osmotica while, during ripening, sugars became predominant. The plots inserted in **Figure 8** show the year-to-year relationships for the six varieties and six microvines reproduced in 2017 (Supplementary Tables S3, S4). Statistical analyses showed a significant effect of genotype, environment and GxE interaction in both genotype subsets for the total content in major osmotica at the ripe stage, but no environmental effect in green fruit for microvines.

### Correlations Between Traits

**FIGURE 9:**
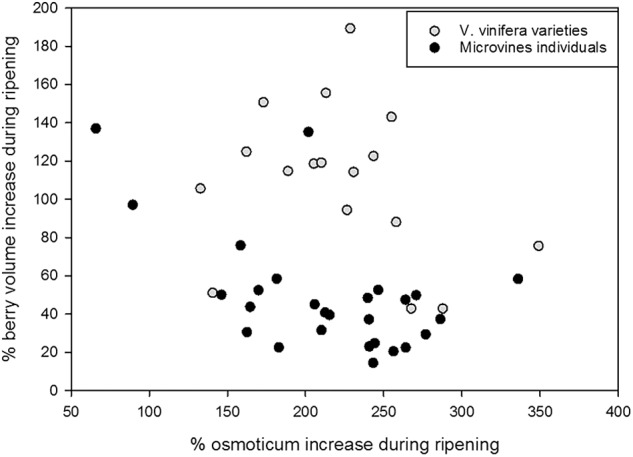
Major osmoticum concentration and fruit volume changes during ripening in varieties (grey dots) and microvine (black dots) subsets. % of osmoticum increase is calculated as 100^∗^(osmoticum contents at max berry growth stage – osmoticum contents in green hard berry)/osmoticum contents in green hard berry. % of berry volume increase is calculated as 100^∗^(berry volume at max berry growth stage – berry volume of green hard berry)/berry volume of green hard berry.

On average, microvines produced smaller berries than varieties. In both subsets, there was no link between fruit volume increase and osmotica content increase (**Figure 9**). In varieties, two significant correlations emerged between fruit traits in varieties at green lag phase: glucose and fructose concentrations (0.60, *p*-value = 1.54 10^-10^), as well as tartaric concentration and fruit volume (-0.56, *p*-value = 3.03 10^-9^). At ripe stage, the only significant correlation was between glucose and fructose concentrations (0.92, *p*-value < 2.2 10^-16^). In microvines, only one significant correlation was found between glucose and fructose concentrations at green lag phase (0.93, *p*-value < 2.2 10^-16^). At ripe stage, glucose and fructose concentrations were correlated (0.98, *p*-value < 2.2 10^-16^) as well as malate concentrations with either glucose (-0.50, *p*-value = 8.49 10^-10^) or fructose (-0.54, *p*-value = 3.03 10^-9^).

**FIGURE 10:**
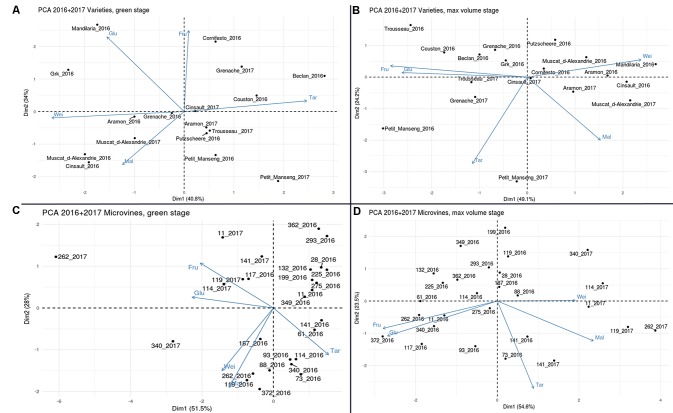
Principal component analyses the all variables collected with varieties **(A,B)** and microvine **(C,D)** subsets, for hard green (left) and ripe (right) berries. Glu (glucose), Fru (Fructose), Tar (tartaric acid), Mal (malic acid) concentrations. Wei (berry weight).

In the PCA analyses (**Figure 10**), the first two principal components explained more than 70% of the phenotypic variability for both subsets and stages. Correlations shown above were represented on PCA plots, which highlighted the low dependence of berry weight on major osmotica. In varieties, Petit Manseng showed a unique localisation with large tartaric acid and sugar concentrations at ripe stage, with a good reproducibility between experiments. For microvines, except for microvines n°141 at ripe stage, PCA suggested a strong environmental effect in relation to the higher size and acidity of the fruits and the lower sugars contents in 2017 as compared to 2016.

## Discussion

### Major Descriptors of Grapevine Fruit Development and Composition

The first critical fruit developmental stage is the green lag phase, which corresponds to the end of the first growing phase when the concentration in organic acids is maximum (Kliewer, 1965; Kliewer et al., 1967). At this stage, berry weight showed a respective 230 and 440% variation among the microvine progeny and varieties. The range observed in the subset of varieties is equivalent to the one reported by Houel et al. (2013) for wine grape cultivars, resulting from the high polymorphism present within the *V. vinifera* variety germplasm (Boursiquot et al., 1995; Fernandez et al., 2006a; Houel et al., 2013). The smaller extend of the berry size diversity observed among microvines can be explained by the intrinsic segregation limitation present in a bi-parental progeny.

The size of the fruit depended on the genotype, the environment and their interaction for varieties, but only on the genotype for microvines, may be because of the little environmental differences between Experiments 3 and 4. In this study, all the genotypes displayed seeded fruit, excluding seedlessness as a potential source for fruit size diversity. In this respect, Houel et al. (2013) demonstrated that seed number or weight did not explain berry growth variation among genotypes. Tartaric and malic acids are major organic acids in *V. vinifera* fruit (Kliewer, 1965; Terrier and Romieu, 2001; Conde et al., 2007; Yinshan et al., 2017). During ripening, tartaric acid concentration decreases by dilution due to fruit enlargement while malic acid concentration decreases through both dilution and respiration (Lakso and Kliewer, 1978; Dai et al., 2011; Famiani et al., 2014). It was previously reported that organic acid concentration and the relative proportions of malate and tartrate varied according to the genotype at ripe stage (Kliewer, 1967a; Liu et al., 2006; Shiraishi et al., 2010). As tartaric acid is not metabolized during ripening (Terrier and Romieu, 2001), its level at the end of green stage is a determinant factor in the final concentration at ripe stage. In this study, the maximum tartaric acid content observed in green berries was 260 meq.L^-1^ for varieties and 180 meq.L^-1^ for microvines, which are higher values than in previous reports (Kliewer et al., 1967; Preiner et al., 2013).

At ripe stage, when the phloem unloading stops, the final quantity of solutes and water per berry determines fruit quality (Matthews et al., 1987; Coombe, 1992; Keller and Shrestha, 2014). Houel et al. (2013) showed that most of the wine varieties displays 1–4 g berries at ripening, which is equivalent to the values observed in this study. Our data confirmed that berry weight at ripe stage varies according to genotype, environment and GxE interactions. For varieties, we observed similar increases in weight between green lag phase and ripe stage in both environments. This suggests that the final fruit size is determined very early during green growth phase. As reported in Houel et al. (2013), fruit weight doubled on average between the herbaceous plateau and ripe stage, but with some extreme behaviors. Indeed, fruit size increment during ripening ranged from x1.4 for Petit Manseng to x2.9 for Cinsaut, suggesting some variability in the control of fruit expansion. For microvines, we found a similar average fruit weight ratio between green and ripe stages in 2017, based on detailed spatial patterns of berry growth. In 2016, we observed smaller fruits at ripe stage for all microvines, but with little impact on the genotype ranking, suggesting a systematic underestimation of the maximum berry volume in Experiment 3.

Regarding the contents in organic acids into *V. vinifera* ripe fruit, Kliewer et al. (1967) reported concentrations ranging from 20 to 100 meq.L^-1^ for malate and from 50 to 100 meq.L^-1^ for tartrate. Using a set of *Vitis* genotypes including interspecific hybrids, Liu et al. (2006) reported a range of 5 meq.L^-1^ to 100 meq.L^-1^ for malate and 20 to 120 meq.L^-1^ for tartrate. Here, we have also identified a huge diversity in the relative abundance of both major organic acids in ripe berries, with a malate to tartrate ratio ranging from 0.13 to 3.62. The sum of concentrations of the two major organic acids in ripe berries ranged from 80 to 361 meq.L^-1^ with respective variations for malate and tartrate from 12 to 276 meq.L^-1^ and 51 to 146 meq.L^-1^, which is larger than previously reported. On average, microvines displayed a higher malic acid concentration at ripe stage than varieties. This can be explained either by genetic or environment effects as microvines were grown in greenhouses, protecting them from pronounced increases in temperature that strongly activate the respiration of malic acid (Kliewer and Lider, 1970; Famiani et al., 2014; Keller, 2015; Rienth et al., 2016b).

During berry ripening sugars progressively become the major osmoticum (Keller, 2015). Among the different sugars accumulated in *V. vinifera* fruits, glucose and fructose are largely dominant (Hawker et al., 1976; Liu et al., 2006; Shiraishi et al., 2010). Famiani et al. (2014) and Keller et al. (2015) confirmed the low quantity of sucrose (<100 mmol.L^-1^; i.e., less than 10% total sugars) in ripe berries with a ratio Glucose/Fructose tending to 1 at ripe stage. Sugar concentration was reported to vary according to environment, cultivation practices and variety (Liu et al., 2007; Dai et al., 2011; Duchêne et al., 2012). Studying 78 genotypes, including table and wine grape cultivars, Kliewer (1967b) reported sugar concentrations ranging from 18.7 (1 mol.L^-1^) to 27 (1.5 mol.L^-1^) °Brix at ripe stage. Recently, Yinshan et al. (2017), reported a huge diversity for sugar concentration in a panel of 45 genotypes, including wine grape varieties from North–East of China. However, these data should be considered with caution due to the imprecision about the stage of sampling. Here, we have observed sugar concentrations ranging from 813 to 1353 mmol.L^-1^ among varieties. This represents a larger range of variation than in most previous studies and corresponds to a slightly lower average value. These differences may be of genetic origin or result from the method used to determine ripe stage. Indeed, when sampling is performed after the maximum berry volume, the concentration of sugar increases by fruit shriveling, even though the quantity of sugar per fruit remains stable. In the microvine progeny, the concentrations of sugars at ripe stage were found lower than in varieties with values ranging from 752 to 1078 mmol.L^-1^.

Low PAR (Photosynthetic Active Radiations) or VPD (Vapor Pressure Deficit) in greenhouses could have reduced leaf carbon assimilation in the greenhouse limiting sugar accumulation flow directed to the fruit despite the source/sink balance was improved by cluster thinning. Moreover, a lower VPD could also be involved in limiting phloem transport of sugars from source organs to the fruit (Keller et al., 2015). Finally, it is also possible that parents of the microvine population carried alleles limiting berry sugar accumulation. Lastly, in previous studies with microvines from various genetic backgrounds, the level of sugar accumulated in berry during ripening was often found to be rather moderate, i.e., 1 mol.L^-1^ or less (Houel et al., 2015; Rienth et al., 2016a; Luchaire et al., 2017). This could indicate that dwarf mutation itself (Boss and Thomas, 2002) or some biological process associated with the dwarf phenotype (Chaib et al., 2010; Torregrosa et al., 2016) are limiting for the accumulation of sugar into the berry.

### Phenotyping at Key Stages of Grapevine Berry Development

The study of the genotypic performances for berry growth and metabolites accumulation needs an accurate protocol to identify key stages of fruit development for each genotype. At the onset of ripening, a cluster is composed of berries with ripening related pathways only activated in a fraction of them (Coombe, 1992; Lund et al., 2008; Gouthu et al., 2014; Rienth et al., 2016b). Similarly, at the end of ripening, the bunch is a mix of berries concentrating primary metabolites by shriveling while other are still importing sugars and water (McCarthy and Coombe, 1999; Shahood, 2017). Because the phenology sequence and berry development asynchronism are both genotype and environment dependent (Costantini et al., 2008; Parker et al., 2011; Doumouya et al., 2014; Rolle et al., 2015; Torchio et al., 2016) it is not possible to predetermine the date of sampling. Duchêne et al. (2012) proposed to compare the genotypic performances in sugar accumulation at defined thermal time points. However, several studies questioned the accuracy of thermal time scaling to study grapevine berry ripening (McIntyre et al., 1987; Rienth et al., 2016b; Romieu et al., 2016). In genetic studies, it is generally not possible to perform a comprehensive fruit sampling sequence for all genotypes since either the time is lacking or the number of fruits is limited. As a consequence of these limitations, almost all genetic studies just described the genotypic diversity at a single stage of berry development, with no precision regarding the real physiological stage of the berry (Preiner et al., 2013; Yinshan et al., 2017).

To offset these limitations and get relevant phenotypic data, we propose to perform the phenotyping at the two stopping phases of berry growth. At the end of the first growth phase, the detection of the first signs of berry softening allows a precise determination of the onset of sugar accumulation (Robin et al., 1997; Abbal et al., 1999; Terrier et al., 2005; Lund et al., 2008; Castellarin et al., 2016). The determination of the end of sugar unloading is more intricate (Doumouya et al., 2014; Shahood et al., 2015; Shahood, 2017). At a berry population level, it has been widely accepted that ripening takes about 40 days after colour change (Mullins et al., 1992), but Costantini et al. (2008) reported ripening periods varying from 10 to 80 days in a *V. vinifera* segregating population. For varieties, which only produce one to three clusters per shoot and reproductive cycle, the monitoring of berry softening during green growth phase allows the selection of clusters with both hard and soft berries. Hard berries can be sampled at that time to represent the very last stages of green berry development. Two or three of these clusters could be further used to non-destructively monitor berry growth by immersion. When the growth of these clusters begins to slow down, a regular sampling of berries on the other synchronized clusters allows the selection of berries at the max fruit volume. For microvines, the best option is to establish controlled conditions of growth to support a continuous and stable reproductive development (Luchaire, 2016). In that case, it is possible to phenotype several stages of development from each plant and use the same plant to harvest successive biological replicates.

### Breeding Prospects

The climate change models (Hannah et al., 2013; Wolkovich et al., 2018) and previous studies on the effect of temperature elevation on grapevine fruit development, provide clues to determine phenotypic targets of future breeding programs. A pre-requisite is to appreciate the magnitude and stability of the fruit trait diversity (Ollat et al., 2014; Gascuel et al., 2017). In this respect, fruit quality at ripe stage result from multi-faced regulatory mechanisms, i.e., metabolite synthesis and degradation together with water accumulation, each one potentially genotype-dependant.

Berry size, that determines fruit yield and quality (Boursiquot et al., 1995; Yamada and Sato, 2016), could be reduced by 30% upon temperature elevation (Kliewer and Lider, 1970; Butrose et al., 1971; Luchaire et al., 2017). Present study underlines how huge the diversity for fruit size is in *V. vinifera* varieties and the possibility to generate new phenotypes by hybridization. This constitutes a favorable context for breeders, even if GxE interactions may disturb the ranking of genotypes.

According to Kliewer and Lider (1970), Butrose et al. (1971), Seguin et al. (2004), and Rienth et al. (2016b), climate warming decrease the acidity of the wines up to 50%, with a marked reduction on malic acid, with already noticeable consequences on wine quality (Escudier et al., 2017). Fruit malic and tartaric acid concentration is depending on genotypic, environmental and GxE interaction effects. Their poor stability and heritability has already impaired the identification of QTLs in grapevine (Chen et al., 2015; Houel et al., 2015). In this study, we observed a huge variability in the contents of malic and tartaric acids and some correlation with berry growth. Thus, among the genotypes characterized here, the larger the berry at ripe stage, the higher the malic acid concentration and the lower the tartaric acid concentration. This explains the high correlation observed between berry size and malic/tartaric acids ratio. The diversity in berry acidity illustrated here represents a smart alternative to present physical or chemical corrections of juice and wine acidity (Escudier et al., 2012; Sweetman et al., 2014). Indeed, European regulations (CEE-606/2009) restrict the supplement of organic acid in grape juices at 20 meq.L^-1^ of either tartaric or malic acid. Moreover, the use of ion exchange resins or bipolar membranes to remove the cations neutralizing organic acids is limited to 54 meq.L^-1^ (CEE 53/2011) for conventional wines and remains prohibited for organic wines is some countries.

Other critical factors for the selection of grapevine fruits better coping with climate warming are the concentration in sugars and the sugars to organic acids ratio (Ojeda et al., 2017a). Kliewer and Lider (1970), Butrose et al. (1971), Rienth et al. (2016a), and Luchaire et al. (2017) showed that temperature elevation could increase sugars concentration up to 3°Brix (0.28 M sugar). Today, European regulations only authorize 20% ethanol removal from the wine (Meillon et al., 2010), which roughly corresponds to 0.12 mol.L^-1^ fruit sugar. Consequently, the genetic diversity for sugar contents observed here and the negative correlation between malic acid and sugar observed here, appear suitable to mitigate the negative impacts of heat on sugar/malic acid ratio (Ojeda et al., 2017b; Torregrosa et al., 2017b). On this respect, the identification of the genetic bases of the extreme phenotypes exhibited in particular by Petit Manseng or Cinsault would provide useful markers for breeding.

## Conclusion

*Vitis vinifera* belongs to an inter-fertile group of species adapted to a diverse range of climates, from hot desert areas to humid tropical regions, which potentially carry valuable reproductive and vegetative traits (Chen et al., 2015; Brillouet et al., 2016; Yamada and Sato, 2016; Koyama et al., 2017). This study highlighted that, despite the high genetic pressure performed on this clonally propagated perennial crop (Zhou et al., 2017; Wolkovich et al., 2018), consistent fruit trait diversity still exists in this taxon. Due to some independence in the segregation of main factors controlling berry growth or primary metabolite accumulation, we also showed that phenotypes with new trait value combinations can be generated by cross-breeding. To be suitable for genetic improvement, phenotypic plasticity must be assessed in a large range of fluctuating environments, a process that remains long and tedious when addressing fruit composition. Fortunately, new genetic resources such as the microvine can boost the identification of fruit traits and their physiological response to abiotic factors (Rienth et al., 2014a,b; 2016b; Luchaire et al., 2017), as well as the discovery of associated QTLs (Chaib et al., 2010; Dunlevy et al., 2013; Houel et al., 2015; Torregrosa et al., 2016, 2017a). Altogether, these observations open prospects for the breeding of varieties with fruit improved in size and composition, to challenge the consequences of climate warming.

## Author Contributions

CR, AB, and LT designed the experiments and drafted the manuscript. AB, DB, EM, and YS performed the experiments. J-MB provided the list of the *V. vinifera* variety subset. LT, AB, CR, J-PP, HO, and AD edited the manuscript. All authors reviewed the final version of the manuscript.

## Conflict of Interest Statement

The authors declare that the research was conducted in the absence of any commercial or financial relationships that could be construed as a potential conflict of interest.

## Footnotes

1 www.oiv.int

2 www.fao.org

3 www.franceagrimer.fr
