# Supplementary material for: Vitis vinifera L. Fruit Diversity to Breed Varieties Anticipating Climate Changes
Source: Front Plant Sci. 2018 May 1;9:455. doi: 10.3389/fpls.2018.00455 (PMC5938353; doi:10.3389/fpls.2018.00455)
Supplement: Supplementary file 1 [file Table_1.PDF]

S1 - Table 1 - List of the genotypes included in the study and their phenotypes for berry size and sugar contents, as proposed by experts or from preliminary experiments.

Genotype name		Variety references Microvines progenitors	Expected berry Size*	Expected sugar concentration**
**Varieties**	Muscat d'Alexandrie	Vass7R08s336-340	Big	High
	Grenache	Vass8R07s001-005	Big	High
	Cinsaut	Vass2R05s551-555	Big	High
	Mandilaria	Vass4R13s596-600	Big	Low
	Grk	Vass4R03s306-310	Big	Low
	Aramon	Vass2R02s046-065	Big	Low
	Plant de Couston	Vass7R12s406-410	Small	High
	Trousseau	Vass2R14s356-360	Small	High
	Petit Manseng	Vass2R12s071-075	Small	High
	Putzscheere	Vass8R02s126-130	Small	Low
	Cornifesto	Vass6R05s001-005	Small	Low
	Béclan	Vass7R11s406-410	Small	Low
**Microvines**	P88	Picovine00C001V0008 x UB *flb*	Big	High
	P114	Picovine00C001V0008 x UB *flb*	Big	High
	P117	Picovine00C001V0008 x UB *flb*	Big	High
	P187	Picovine00C001V0008 x UB *flb*	Big	High
	P262	Picovine00C001V0008 x UB *flb*	Big	High
	P28	Picovine00C001V0008 x UB *flb*	Big	Low
	P61	Picovine00C001V0008 x UB *flb*	Big	Low
	P119	Picovine00C001V0008 x UB *flb*	Big	Low
	P132	Picovine00C001V0008 x UB *flb*	Big	Low
	P293	Picovine00C001V0008 x UB *flb*	Big	Low
	P340	Picovine00C001V0008 x UB *flb*	Big	Low
	P349	Picovine00C001V0008 x UB *flb*	Big	Low
	P11	Picovine00C001V0008 x UB *flb*	Small	High
	P73	Picovine00C001V0008 x UB *flb*	Small	High
	P93	Picovine00C001V0008 x UB *flb*	Small	High
	P141	Picovine00C001V0008 x UB *flb*	Small	Low
	P199	Picovine00C001V0008 x UB *flb*	Small	Low
	P225	Picovine00C001V0008 x UB *flb*	Small	Low
	P275	Picovine00C001V0008 x UB *flb*	Small	Low
	P362	Picovine00C001V0008 x UB *flb*	Small	Low
	P372	Picovine00C001V0008 x UB *flb*	Small	Low

* Big berries (> 1.5 g), ** High sugar contents (> 1 mol/L-1)
