# Supplementary material for: Vitis vinifera L. Fruit Diversity to Breed Varieties Anticipating Climate Changes
Source: Front Plant Sci. 2018 May 1;9:455. doi: 10.3389/fpls.2018.00455 (PMC5938353; doi:10.3389/fpls.2018.00455)
Supplement: Supplementary file 2 [file Table_2.PDF]

S2 - Table 2 - Sum of the GDD (growing degree days) in base 10 and means of the maximum temperatures measured during the 4 months of sampling in field (Exp 1 and 2) and greenhouse (Exp 3 and 4).

**Subset**	**Varieties (Field)**				**Microvines (Greenhouse)**			
Experiment	Exp 1 (2016)		Exp 2 (2017)		Exp 3 (2016)		Exp 4 (2017)	
Month	GDD10	Avr Tmax	GDD10	Tmax	GDD10	Tmax	GDD10	Tmax
June	337	25.4	419	29.9	467	32.7	374	28.4
July	428	29,0	464	30.8	474	29,0	394	29.5
August	407	28.2	455	30.6	448	27.1	390	29.1
September	347	26.7	265	25,0	408	27.9	332	28.3
**Sum*/Avr****	**1520**	**27.3**	**1603**	**29.1**	**1796**	**29.2**	**1490**	**28.8**

* Sum of the GDD10 for the 4 months of fruit ripening. ** Average of the maximum air T°
