# Supplementary material for: Vitis vinifera L. Fruit Diversity to Breed Varieties Anticipating Climate Changes
Source: Front Plant Sci. 2018 May 1;9:455. doi: 10.3389/fpls.2018.00455 (PMC5938353; doi:10.3389/fpls.2018.00455)
Supplement: Supplementary file 3 [file Table_3.PDF]

S3 - Table 3 - Mean values of the fruit parameters measured for the six V. vinifera varieties experimented in years 2016 (Exp. 1) and 2017 (Exp 2).

Stage of berry development	Variety	Experiment	FW* Mean	FW* SE		M+T** Mean	M+T** SE		Tartrate** Mean	Tartrate** SE		M/T** Mean	M/T** SE		G+F*** Mean	G+F*** SE		Osmotica*** Mean	Osmotica*** SE	
**Green plateau**	Aramon	N°1 (2016)	1.4	*0.3*	def	497	*26*	bc	140	*7*	a	2.6	*0.3*	de	118	*6*	d	366	*16*	cd
		N°2 (2017)	1.1	*0.0*	cde	403	*18*	a	152	*6*	abc	1.6	*0.0*	ab	87	*4*	abc	289	*13*	a
	Cinsaut	N°1 (2016)	1.9	*0.3*	f	605	*12*	de	142	*11*	a	3.3	*0.4*	f	102	*20*	bc	404	*20*	e
		N°2 (2017)	0.9	*0.1*	abc	487	*42*	bc	164	*8*	abc	2.0	*0.1*	bc	114	*2*	cd	358	*19*	cd
	Grenache	N°1 (2016)	1.0	*0.2*	bcd	467	*12*	ab	149	*8*	ab	2.1	*0.1*	cd	115	*16*	cd	348	*15*	c
		N°2 (2017)	0.7	*0.1*	a	424	*115*	ab	175	*46*	abc	1.4	*0.0*	a	128	*47*	bcd	340	*31*	abc
	Muscat d'Alexandrie	N°1 (2016)	2.4	*0.2*	g	539,8	*11*	c	147	*9*	ab	2.7	*0.2*	e	98	*15*	bc	368	*13*	cd
		N°2 (2017)	1.8	*0.0*	efg	494	*34*	bc	156	*12*	abc	2.2	*0.1*	cde	101	*7*	bcd	348	*23*	bc
	Petit Manseng	N°1 (2016)	0.9	*0.1*	ab	614,8	*30*	e	175	*18*	c	2.6	*0.4*	e	86	*19*	ab	394	*19*	de
		N°2 (2017)	0.8	*0.0*	a	515	*81*	cd	185	*27*	bc	1.8	*0.1*	abc	45	*6*	a	303	*35*	ab
	Effect *p*-values	Genotype	$< 2.2\ 10^{-16}$			$4.22\ 10^{-16}$			$5.04\ 10^{-4}$			$1.89\ 10^{-11}$			$1.12\ 10^{-4}$			$5.48\ 10^{-7}$		
		Environment	$1.12\ 10^{-9}$			$1.22\ 10^{-10}$			$9.41\ 10^{-6}$			$< 2.2\ 10^{-16}$			0.157			$5.03\ 10^{-11}$		
		G x E	$1.12\ 10^{-3}$			$2.42\ 10^{-2}$			$3.78\ 10^{-1}$			$4.77\ 10^{-3}$			$9.48\ 10^{-5}$			$5.30\ 10^{-5}$		
**Ripe fruit**	Aramon	N°1 (2016)	3.6	*0.4*	g	140	*21*	bcde	74	*3*	bc	0.9	*0.2*	de	931	*43*	ab	999	*35*	ab
		N°2 (2017)	2.3	*0.1*	a	141,4	*10*	bcd	102	*7*	ef	0.4	*0.0*	ab	885	*61*	a	955	*66*	a
	Cinsaut	N°1 (2016)	4.3	*0.4*	fg	153,3	*15*	cde	63	*3*	a	1.4	*0.2*	f	983	*49*	abc	1059	*42*	abc
		N°2 (2017)	2.7	*0.2*	e	139,7	*5*	cd	81	*2*	cd	0.7	*0.1*	d	1105	*37*	cde	1175	*36*	cd
	Grenache	N°1 (2016)	2.5	*0.0*	e	109,1	*12*	ab	70	*2*	ab	0.6	*0.2*	bcd	1180	*100*	def	1235	*94*	cde
		N°2 (2017)	1.4	*0.1*	cd	151	*6*	cde	106	*3*	f	0.4	*0.1*	bc	1142	*64*	def	1217	*63*	cde
	Muscat d'Alexandrie	N°1 (2016)	5.2	*0.7*	f	127,8	*9*	bc	72	*2*	ab	0.8	*0.2*	de	1076	*26*	bcde	1140	*22*	bcd
		N°2 (2017)	3.9	*0.2*	g	165,9	*19*	de	84	*13*	d	1.0	*0.1*	e	925	*164*	a	1006	*170*	ab
	Petit Manseng	N°1 (2016)	1.3	*0.3*	bcd	187	*14*	ef	147	*9*	g	0.3	*0.0*	a	1353	*128*	f	1447	*126*	de
		N°2 (2017)	1.1	*0.0*	b	243	*6*	f	144	*5*	g	0.7	*0.0*	d	1052	*40*	bcd	1174	*43*	cd
	Trousseau	N°1 (2016)	1.3	*0.1*	d	80	*0*	a	62	*2*	a	0.3	*0.1*	a	1343	*57*	f	1383	*57*	e
		N°2 (2017)	1.5	*0.1*	c	141	*10*	cd	86	*5*	de	0.6	*0.0*	cd	1208	*82*	ef	1279	*87*	e
	Effect *p*-values	Genotype	$< 2.2\ 10^{-16}$			$2.92\ 10^{-12}$			$3.93\ 10^{-15}$			$8{,}05\ 10^{-10}$			$1.73\ 10^{-10}$			$7.71\ 10^{-12}$		
		Environment	$1.52\ 10^{-15}$			$4.48\ 10^{-5}$			$1.58\ 10^{-13}$			0.33			$3.66\ 10^{-3}$			$8.97\ 10^{-3}$		
		G x E	$1.73\ 10^{-4}$			$3.12\ 10^{-4}$			$4.42\ 10^{-8}$			$2.43\ 10^{-15}$			$9.75\ 10^{-6}$			$4.08\ 10^{-4}$		

* FW, Fresh weight in g, ** M+T (Malate+Tartrate), M/T (Malate/tartrate) and Tartrate in meq.L-1, *** G+T (Glucose + Fructose) and Sum of major osmotica (M+T+G+F) in mmol.L-1
