# Supplementary material for: Vitis vinifera L. Fruit Diversity to Breed Varieties Anticipating Climate Changes
Source: Front Plant Sci. 2018 May 1;9:455. doi: 10.3389/fpls.2018.00455 (PMC5938353; doi:10.3389/fpls.2018.00455)
Supplement: Supplementary file 4 [file Table_4.PDF]

S4 - Table 4 - Mean values of the fruit parameters measured for the six microvine offspring experimented in years 2016 (Exp 3) and 2017 (Exp 4).

Stage of berry development	Genotype	Experiment	FW* Mean	FW* *SE*		M+T** Mean	M+T** *SE*		Tartrate** Mean	Tartrate** *SE*		M/T** Mean	M/T** *SE*		G+F*** Mean	G+F*** *SE*		Osmotica*** Mean	Osmotica*** *SE*	
Green plateau	011	N°3 (2016)	1.3	*0.3*	abc	429	*18*	ab	137.8	*6*	d	2.1	*0.1*	a	34.7	*11*	abcd	249	*16*	ab
		N°4 (2017)	1.4	*0.3*	abc	402	*85*	a	111.6	*19*	bc	2.6	*0.4*	b	78.3	*69*	d	279	*104*	ab
	114	N°3 (2016)	1.5	*0.1*	bc	542	*34*	bcde	121.9	*13*	cd	3.5	*0.4*	cd	15.2	*5*	a	286	*15*	ab
		N°4 (2017)	1.5	*0.2*	bc	481	*131*	abcd	93.9	*19*	ab	4.1	*0.5*	de	52.3	*34*	cd	293	*92*	ab
	119	N°3 (2016)	1.7	*0.2*	bc	599	*59*	e	122.3	*10*	cd	3.9	*0.4*	de	38.6	*12*	abcd	338	*31*	bc
		N°4 (2017)	1.6	*0.4*	bc	424	*158*	a	92.9	*43*	a	3.7	*0.4*	de	43.6	*26*	bcd	255	*100*	a
	141	N°3 (2016)	1.3	*0.1*	ab	482	*49*	abcde	129.4	*15*	cd	2.8	*0.8*	ab	16.8	*10*	a	258	*34*	ab
		N°4 (2017)	1.2	*0.1*	a	436	*101*	abc	109.2	*24*	bc	3.0	*0.4*	bc	53.8	*27*	cd	272	*68*	ab
	262	N°3 (2016)	1.8	*0.2*	c	560	*19*	cde	115.0	*14*	bc	3.9	*0.5*	de	29.1	*9*	abc	309	*30*	ab
		N°4 (2017)	1.6	*0.5*	bc	565	*55*	de	81.7	*15*	a	6.1	*0.9*	f	153.3	*84*	e	436	*86*	c
	340	N°3 (2016)	1.4	*0.2*	abc	586	*21*	de	123.9	*5*	cd	3.7	*0.3*	de	19.9	*5*	ab	313	*9*	ab
		N°4 (2017)	1.4	*0.1*	abc	644	*45*	bcde	108.3	*1*	abc	4.9	*0.4*	ef	89.1	*22*	cde	411	*45*	bc
	Effect p-values	Genotype	$3.38\ 10^{-9}$			$5.26\ 10^{-8}$			$1.77\ 10^{-8}$			$< 2.2\ 10^{-16}$			$1.18\ 10^{-7}$			$5.4\ 10^{-8}$		
		Environment	0.09			0.039			$8.46\ 10^{-14}$			$8.52\ 10^{-9}$			$7.18\ 10^{-13}$			0.13		
		G x E	0.46			8.76 10-3			$6.14\ 10^{-1}$			$1.72\ 10^{-4}$			$2.24\ 10^{-3}$			$1.66\ 10^{-4}$		
Ripe fruit	011	N°3 (2016)	1.8	*0.3*	ab	146	*13*	a	87.8	*9*	bc	0.7	*0.1*	a	889	*36*	cd	962	*35*	bc
		N°4 (2017)	1.8	*0.2*	abc	250	*68*	c	92.3	*12*	bc	1.7	*0.6*	bcd	609	*122*	ab	733	*89*	a
	114	N°3 (2016)	2.1	*0.1*	bc	152	*7*	ab	78.0	*10*	ab	1.0	*0.3*	ab	827	*87*	bc	903	*86*	ab
		N°4 (2017)	2.6	*0.1*	de	256	*52*	c	73.3	*12*	ab	2.5	*0.1*	cd	629	*28*	a	757	*46*	a
	119	N°3 (2016)	2.6	*0.2*	de	147	*18*	a	60.7	*6*	a	1.4	*0.3*	b	760	*32*	abc	833	*28*	ab
		N°4 (2017)	3.7	*1.0*	e	260	*81*	bcd	101.0	*21*	bc	1.7	*1.4*	abcd	641	*147*	abc	771	*107*	ab
	141	N°3 (2016)	1.6	*0.2*	a	247	*68*	c	96.9	*26*	bc	1.6	*0.3*	bc	763	*74*	abc	887	*61*	ab
		N°4 (2017)	1.5	*0.2*	a	269	*38*	cd	114.7	*6*	c	1.4	*0.4*	bc	709	*153*	abc	844	*134*	ab
	262	N°3 (2016)	2.2	*0.2*	cd	173	*34*	abc	69.7	*10*	ab	1.5	*0.3*	b	1014	*55*	e	1101	*39*	d
		N°4 (2017)	3.2	*0.6*	de	362	*33*	d	84.8	*25*	abc	3.6	*1.6*	d	644	*260*	abcd	825	*250*	abcd
	340	N°3 (2016)	1.9	*0.2*	abc	187	*28*	abc	79.0	*10*	b	1.4	*0.4*	b	972	*38*	de	1066	*46*	cd
		N°4 (2017)	3.4	-	e	152	-	abc	71.7	-	abc	1.1	-	ab	605	-	ab	681	-	a
	Effect p-values	Genotype	$4.81\ 10^{-13}$			$3.16\ 10^{-4}$			$2.71\ 10^{-4}$			$3.20\ 10^{-4}$			$5.79\ 10^{-6}$			$1.19\ 10^{-5}$		
		Environment	$3.80\ 10^{-5}$			$5.62\ 10^{-10}$			$3.25\ 10^{-2}$			$9.36\ 10^{-6}$			$6.17\ 10^{-10}$			$6.37\ 10^{-8}$		
		G x E	$1.60\ 10^{-4}$			$3.35\ 10^{-4}$			$2.70\ 10^{-2}$			$2.95\ 10^{-5}$			$1.33\ 10^{-2}$			$1.61\ 10^{-2}$		

* FW, Fresh weight in g, ** M+T (Malate+Tartrate), M/T (Malate/tartrate) and Tartrate in meq.L-1, *** G+T (Glucose + Fructose) and Sum of major osmotica (M+T+G+F) in mmol.L-1
